# Efficacy and safety of PD‐1/PD‐L1 inhibitors in elderly patients with advanced non‐small cell lung cancer

**DOI:** 10.1111/crj.13763

**Published:** 2024-05-08

**Authors:** Li Li, Chunhua Xu, Wei Wang, Qian Zhang

**Affiliations:** ^1^ Department of Respiratory Medicine The Affiliated Nanjing Brain Hospital of Nanjing Medical University Nanjing China; ^2^ Clinical Center of Nanjing Respiratory Diseases and Imaging Nanjing China

**Keywords:** efficacy, elderly patients, immunotherapy, non‐small cell lung cancer, PD‐1/PD‐L1 inhibitors, safety

## Abstract

**Objective:**

This study aimed to investigate the efficacy and safety of PD‐1/PD‐L1 inhibitors in treatment of elderly patients with advanced non‐small cell lung cancer (NSCLC).

**Methods:**

Patients with advanced NSCLC ≥70 years old who received PD‐1/PD‐L1 inhibitors in our hospital were retrospectively analyzed. According to age, the patient were stratified as follows: 70–75 years old, 76–80 years old, and >80 years old. Kaplan–Meier method was used for survival analysis, and univariate and multivariate Cox proportional hazards regression models were used to analyze the correlation between different clinical characteristics and survival.

**Results:**

A total of 58 elderly patients with advanced non‐small cell cancer were enrolled in this study. Patients aged 70–75, 76–80, and >80 years old were 32, 19, and 7, respectively. For the all, median OS was 17.0 months, and PFS was 7.0 months. PFS and OS did not differ according to age (*P* = 0.396, 0.054, respectively). Univariate analysis showed that PS of 0–1, stage III, first‐line therapy and irAEs were associated with longer PFS, and PS of 0–1, stage III, and first‐line therapy were associated with longer OS. Multivariate analysis showed that patients with stage III had longer PFS. PFS and OS of patients with PS ≥ 2 were significantly shorter than those of patients with PS of 0–1.

**Conclusions:**

In the present real‐world retrospective cohort, PD‐1/PD‐L1 inhibitors are effective and well tolerated in elderly patients with advanced NSCLC. Immunotherapy should be actively used as early as possible in older patients advanced NSCLC.

## Graphical abstract

With the increasing age of the global population, the number of elderly patients with lung cancer is increasing. Therefore, it is essential to analyze the efficacy and safety of immunotherapy in elderly patients. Our study indicates that immunotherapy will become the main treatment for elderly patients with advanced NSCLC.

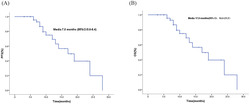

## 1 INTRODUCTION

Lung cancer is one of the most common and deadly types of malignant tumors worldwide.^1^ Non‐small cell lung cancer (NSCLC) is the second most common malignant tumor in the world. In recent years, with the aging of the population, the incidence of lung cancer in the elderly is increasing, and about half of the lung cancer patients are over 70 years old. The incidence is expected to continue to increase in the coming decades.^2^ A number of studies have proved that elderly patients can derive similar survival benefits as young patients when treated with chemotherapy.^3^, ^4^

In recent years, with the development of molecular biology and immunology, immunotherapy and targeted therapy have become the main treatment for lung cancer, and immunotherapy is the standard treatment for non‐small cell lung cancer without gene mutations. Several clinical trials have shown that compared with chemotherapy, immunotherapy can improve the survival and quality of patients, and the side effects are tolerable.^5^, ^6^, ^7^, ^8^ Because changes in physiological functions related to aging may affect the pharmacokinetics and pharmacodynamics of drugs, these elderly patients are excluded from many pivotal clinical trials, making the treatment of elderly patients with lung cancer challenging.^9^ Therefore, it is essential to analysis the efficacy and safety of immunotherapy in elderly patients. Currently, 70 years old has been used in clinical trials specifically targeting older populations.^10^ To this aim, we performed a retrospective analysis of 58 advanced NSCLC patients over 70 years old who received PD‐1/PD‐L1 inhibitors.

## 2 MATERIAL AND METHODS

### 2.1 Patient population and data collection

The clinical data of advanced NSCLC patients aged ≥70 years who treated with PD‐1/PD‐L1 inhibitors in our hospital from October 2019 to June 2022 were retrospectively collected. The inclusion criteria were as follows: cytological and/or pathological confirmed locally advanced or advanced non‐small cell lung cancer (stage IIIB or IV), age ≥70 years old, and receiving at least one cycle of PD‐1/PD‐L1 inhibitor. Data were retrieved from electronic patient records by professional clinicians. Age, sex, tobacco status, PS score, histological type, TNM stage, mutation status, treatment line, treatment protocol, and immune‐related adverse events (irAEs) were collected. All patients signed informed consent for the study.

### 2.2 Assessments

Tumor response was assessed every 4–8 weeks through CT or MRI scans, according to the Response Evaluation Criteria in solid tumors (RECIST version 1.1). Based on this, the efficacy included CR, PR, SD, and PD. Objective response rate (ORR) was defined as the sum of CR and PR, while disease control rate (DCR) included CR, PR, and SD. Progression‐free survival (PFS) was defined as the time from the treatment start to disease progression or death (event). Overall survival (OS) was defined as the beginning of immunotherapy to death from any cause. The Common Terminology Criteria for Adverse Events (CTCAE) was used to assess immune‐related adverse events (irAEs) which reflects the disorder of the immune system.

### 2.3 Statistical analysis

SPSS 26.0 software was used for the all statistical analysis. Kaplan–Meier method was used to analyze PFS and OS survival curves. Cox proportional hazard regression model was used to evaluate the association between different clinical characteristics and survival. *P* < 0.05 was considered statistically significant.

## 3 RESULTS

### 3.1 Patient and disease characteristics

A total of 58 patients were enrolled in the present study. Patients aged 70–75, 76–80, and >80 years old were 32, 19, and 7, respectively. Table 1 summarizes the characteristics of patients. The age ranged from 70 to 90 years old, and the median age is 76. There were 47 males (81.0%) and 51 females (19.0%). There were 42 patients (72.4%) with smoking history and 16 patients (27.6%) without smoking history. Forty‐nine patients (84.5%) had PS of 0–1, and 9 patients (15.5%) had PS ≥ 2. There were 9 cases (15.5%) of stage III and 49 cases (84.5%) of stage IV. Thirty‐six (62.1%) patients were diagnosed as squamous cell carcinoma, and non‐squamous carcinoma mainly included adenocarcinoma, mixed type and undetermined type. The 8 patients (6.9%) with EGFR mutation were treated with molecular‐targeted drugs as first‐line treatment, and after resistance to targeted therapy, immunotherapy is selected as second or more line treatment. There were 54 patients (93.1%) with EGFR mutation negative or unknown. Twenty‐eight patients (48.3%) used PD‐1/PD‐L1 inhibitors as the first‐line treatment, and 30 patients (51.7%) used PD1/PD‐L1 inhibitors as the second line or more. The majority of patients (49 cases, 84.5%) were treated with combination therapy, including immunotherapy combined with chemotherapy, immunotherapy combined with anti‐angiogenesis, and immunotherapy combined with chemotherapy and anti‐angiogenesis. Only 9 patients were treated with monotherapy. Fourteen patients had irAEs of any grade (24.1%) during immunotherapy, mainly included pneumonitis, dermatitis, myelosuppression, hypothyroidism, hyperthyroidism, and myasthenia, which were improved after symptomatic treatment.

**TABLE 1:** Characteristics of patients.

Characteristics	*N*	%
Sex		
Male	47	81.0%
Female	11	19.0%
Smoking history		
Ever/current	42	72.4%
Never	16	27.6%
ECOG PS		
0–1	49	84.5%
≥2	9	15.5%
TNM stage		
IIIB	9	15.5%
IV	49	84.5%
Histological type		
Squamous cell	36	62.1%
Non‐squamous cell	22	37.9%
EGFR mutation		
Mutant	4	6.9%
Wild or unknown	54	93.1%
Treatment line		
First line	28	48.3%
Others	30	51.7%
Immunotherapy protocols		
Monotherapy	9	15.5%
Combination therapy	49	84.5%
irAEs		
Yes	14	24.1%
No	44	75.9%

### 3.2 Outcome analysis

**FIGURE 1:**
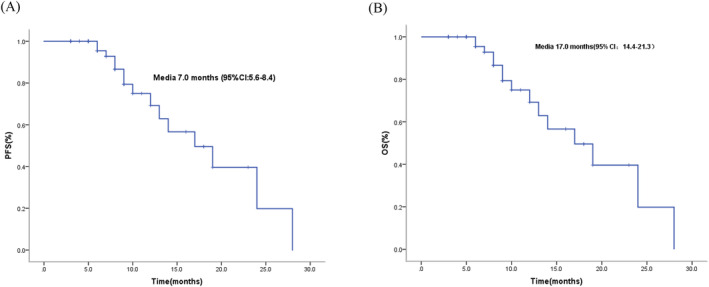
Kaplan–Meier curves for overall PFS and OS.

**FIGURE 2:**
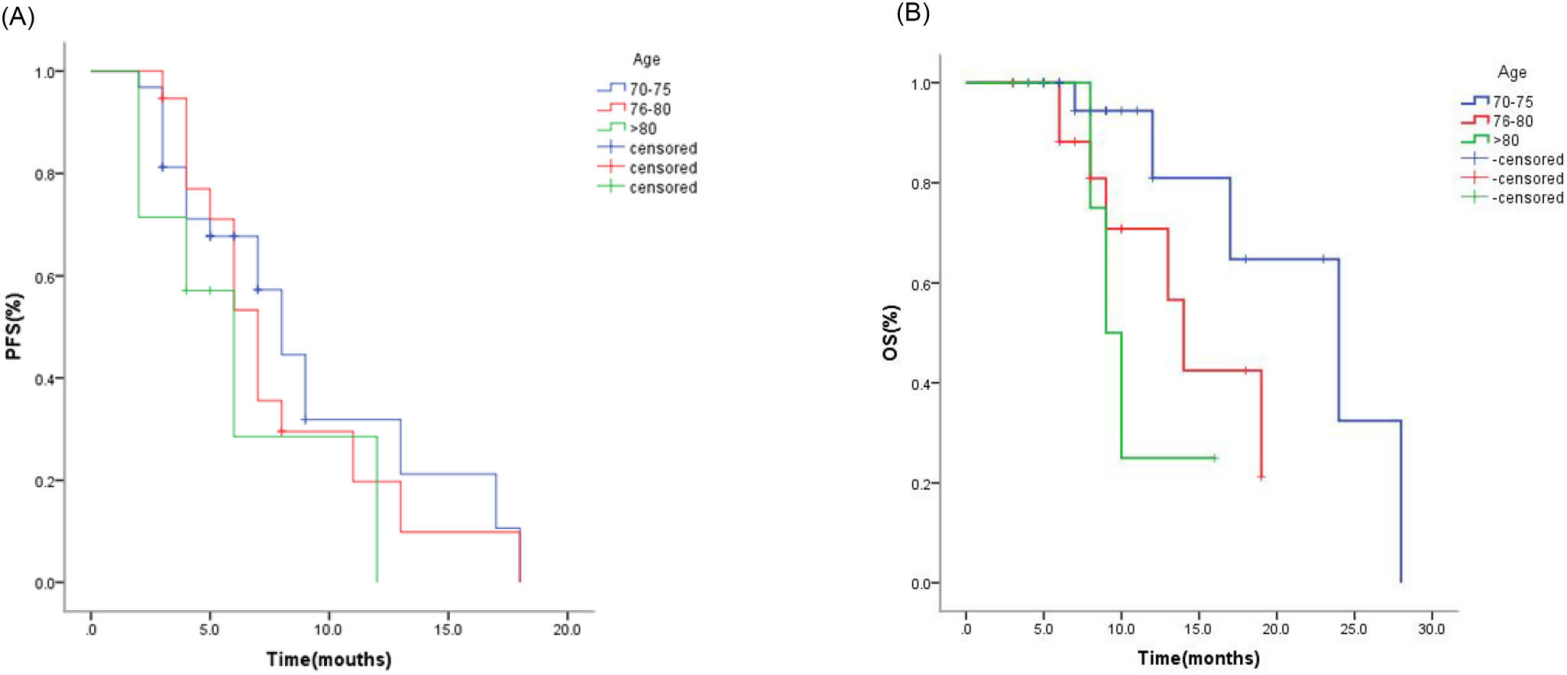
Kaplan–Meier curves for PFS and OS according to age classes.

For the population overall, no patients achieved complete response, 17 (29.3%) patients showed partial response, 33 (56.9%) patients presented stable disease, and 8 (13.8%) patients developed progressive disease. An overall disease control rate (DCR) was obtained in 50 patients (86.2%), while the objective response rate (ORR) was 29.3%. When stratified according to age, there was no statistical difference in ORR between the three classes (31.2% vs. 26.3% vs. 28.6%, *P* > 0.05). For the global population, the median PFS of was 7.0 months (95% CI: 5.6–8.4), and median OS was 17.0 months (95% CI: 10.2–23.8) (Figure 1). According to age, there were differences in PFS and OS, the median PFS of patients aged 70–75 years old was 8.0 months, 7.0 months for patients aged 76–80, and for aged >80, PFS was 6.0 months (*P* = 0.396). Median OS of patients aged 70–75 years old was 24.0 months; for aged 76–80, OS was 14.0 months and 9.0 months for aged >80 (*P* = 0.054) (Figure 2).

Univariate analysis confirmed that PS score of 0–1 (*P* < 0.001), stage III (*P* = 0.009), first‐line therapy (*P* = 0.014), and irAEs (*P* = 0.034) showing an impact on PFS. At multivariate analysis, the single parameter significantly associated with PFS were the PS score (HR = 5.057, 95% CI: 1.875–13.639, *P* = 0.001) and TNM stage (HR = 11.206, 95% CI: 1.369–91.688, *P* = 0.024) (Table 2). The median OS was 17.0 months (95% CI: 10.2–23.8) (Figure 2). The positive impact of PS ≤ 1 on OS was also identified in both univariate and multivariate analyses. Univariate analysis confirmed PS of 0–1 (*P* = 0.003), stage III (*P* = 0.024), and first‐line therapy (*P* = 0.048) associated with OS; further multivariate analysis showed that PS score (HR = 5.861, 95% CI: 1.199–28.645, *P* = 0.029) had a statistically significant effect on OS (Table 3).

**TABLE 2:** Univariate and multivariate analysis for PFS.

Factors	Univariate analysis		Multivariate analysis	
	HR (95% CI)	*P*	HR (95% CI)	*P*
Gender Male vs. female	1.011 (0.438–2.334)	0.980		
Smoking history Ever/current vs. never	1.044 (0.526–2.073)	0.902		
ECOG PS 0–1 vs. ≥2	6.015 (2.248–16.096)	<0.001	5.057 (1.875–13.639)	0.001
TNM stage IIIB vs. IV	15.149 (1.989–115.361)	0.009	11.206 (1.369–91.688)	0.024
Histological type Squamous vs. non‐squamous	1.619 (0.790–3.319)	0.188		
EGFR mutation Mutant vs. wild or unknown	0.790 (0.188–3.309)	0.747		
Treatment line First line vs. others	2.525 (1.210–5.270)	0.014	1.780 (0.832–3.807)	0.137
Immunotherapy protocols Monotherapy vs. combination	0.737 (0.324–1.731)	0.484		
irAEs Yes vs. no	2.470 (1.070–5.702)	0.034	1.405 (0.573–3.447)	0.458

**TABLE 3:** Univariate and multivariate analysis for OS.

Factors	Univariate analysis		Multivariate analysis	
	HR (95% CI)	*P*	HR (95% CI)	*P*
Gender Male vs. female	1.325 (0.364–4.833)	0.669		
Smoking history Ever/current vs. never	1.910 (0.640–5.705)	0.246		
ECOG PS 0–1 vs. ≥2	8.530 (2.081–34.958)	0.003	5.861 (1.199–28.645)	0.029
TNM stage IIIB vs. IV	6.143 (1.263–29.880)	0.024	5.094 (0.943–27.527)	0.059
Histological type Squamous vs. non‐squamous	1.630 (0.466–5.697)	0.444		
EGFR mutation Mutant vs. wild or unknown	1.109 (0.142–8.692)	0.921		
Treatment line First line vs. others	3.318 (1.012–10.877)	0.048	1.238 (0.310–4.924)	0.763
Immunotherapy protocols Monotherapy vs. combination	1.328 (0.390–4.524)	0.650		
irAEs Yes vs. no	2.694 (0.735–9.879)	0.135		

### 3.3 Analysis of immune‐related adverse events

**FIGURE 3:**
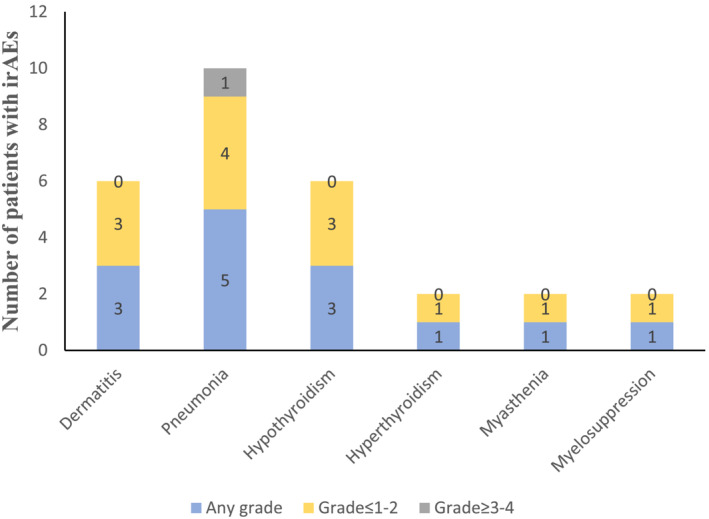
Immune‐related adverse events observed during immunotherapy.

A total of 14 patients experiencing immune‐related adverse events (irAEs), included pneumonia, dermatitis, bone marrow suppression, hypothyroidism, hyperthyroidism, and myasthenia. The most common irAEs was pneumonia (5, 8.6%), 3 (5.2%) patients presented dermatitis, and 3 (5.2%) had hypothyroidism. Other irAEs were hyperthyroidism (1, 1.7%), myelosuppression (1, 1.7%), and myasthenia (1, 1.7%). All irAEs were grade 1–2 except for one patients of grade 3 pneumonitis. No deaths associated with irAEs were observed in this study (Figure 3).

## 4 DISCUSSION

Studies have proven that aging can lead to some structural and functional changes of the immune system, which are defined as “immunosenescence.” Immunosenescence is not simply a gradual decline of all immune functions, but a dynamic remodeling and adaptation process. “Immunosenescence” may predict the efficacy of immunotherapy in elderly NSCLC patients.^11^ An experiment on mice showed that the number of T cells is reduced and the proliferation rate of antigen‐stimulated lymphocytes slowed down in aged mice, when mice are exposed to ICIs, the oxidative capacity of aged mice is increased, the release of proinflammatory mediators is uncontrolled, and the risk of serious immune‐related adverse events is increased in aged mice compared with young mice.^12^ With the aging of the population, elderly patients are the main population of lung cancer patients, but they are excluded from many clinical trials including immunotherapy because of their special physiological functions. Elderly patients who participate in clinical trials are usually healthier than those who are actually treated in clinical practice, and their data are generally obtained through subgroup analysis which lack of relevant data demonstration in the real world. Hence, the efficacy and safety in elderly patients treated with immunotherapy in the real world still need to be further confirmed.

In recent years, research on immunotherapy in real‐world elderly patients has attracted the attention of investigators worldwide. In a study of squamous NSCLC receiving nivolumab, investigators divided patients into <65, 65–75, and >75 years, which showed that among the three groups, there was no statistical difference in RR and PFS and well tolerated in all age groups. The group of 75 years old had a shorter OS; death may have been due to other complications. The results suggest that elderly patients with advanced squamous NSCLC can benefit from nivolumab treatment, and their tolerance is similar to that of the general population.^13^ Giulia et al. also suggested that advanced age was not related to immunotherapy, and even in the oldest patients, there were no intolerable toxic reactions. Researchers believe that immunotherapy should be considered in all elderly patients as long as they have good PS, and immunotherapy is usually the only treatment option for elderly patients who cannot tolerate chemotherapy.^14^ A meta‐analysis of 12 clinical studies showed that ICIs improved OS in both the elderly and young groups, but patients older than 75 years did not show any significant benefit from immunotherapy compared with patients aged 65–75 years. Poor tolerance to treatment may therefore affect the efficacy of immunotherapy.^15^

This study showed that PD‐1/PD‐L1 inhibitors showed better clinical efficacy in elderly patients, with a DCR of 86.2% and an O of 29.3%. A study on elderly patients and young patients receiving immunotherapy analyzed the efficacy of immunotherapy for patients aged <60, 60–74, and ≥75, respectively; the results showed that the ORRs of the three groups were 33.3%, 52.8%, and 53.3%, respectively, and the DCR were 81.0%, 84.3% and 100.0%, respectively. Even the elderly patients aged ≥75 could also achieve good feasibility.^16^ Cox analysis showed that patients with PS 0–1 had better PFS and OS than patients with PS 2. PS reflects the patient's physical status and ability to perform daily activities. PS ≥ 2 is associated with poor prognosis. Elderly patients with a good PS have better tolerance to immunotherapy and other treatments. Multivariate analysis showed that PS was an independent prognostic factor. Therefore, elderly patients with poor PS are not suitable for immunotherapy, especially those with multiple comorbidities. After years of exploration and clinical research, immunotherapy is considered to be the first‐line treatment without gene mutations. Studies have shown that the efficacy of first‐line immunotherapy is significantly better than that of second‐line and above treatment, whether it is s monotherapy or immunotherapy combined with chemotherapy.^17^, ^18^ Compared with second line and further, first‐line immunotherapy shows better OS and PFS. Patients who receive immunotherapy in the first line usually have a better immune status, while those who have previously received chemotherapy may have their immune system damaged. Therefore, elderly patients should also receive immunotherapy as early as possible.

A number of previous studies indicated that the presence of irAEs is related with the improvement of treatment efficacy in NSCLC patients receiving ICIs treatment.^19^, ^20^ The univariate analysis results of this study showed that irAEs in elderly patients had better PFS, but had no correlation with OS, which may be related to drug discontinuation due to irAEs. The safety of immunotherapy in elderly patients has attracted much attention because of the changes in physiological function of elderly patients. In this study, the adverse reactions of immunotherapy in elderly patients were generally mild and no fatal side effects were observed.

In conclusion, through the analysis of real world data, our study shows that immunotherapy has good feasibility in elderly age patients with advanced NSCLC. It indicates that age may not the main factor for clinicians to consider whether to use immunotherapy for elderly NSCLC patients, and immunotherapy will become the main treatment for elderly patients. If patients can tolerate, early application of immunotherapy may be more beneficial in clinical practice.

Of course, this study also has certain limitations. First, this study is a retrospective study with a certain selection bias inevitably. Second, it is a single arm study, and patients receiving non immunotherapy are not included as the control group. In addition, the sample size of this study is limited and subgroup analysis cannot be conducted. In the future, large‐sample prospective studies are needed to verify the efficacy and safety of PD‐1/PD‐L1 inhibitors in elderly patients with NSCLC.

## AUTHOR CONTRIBUTIONS

Chunhua Xu designed the study. Wei Wang and Qian Zhang collected data. Li Li wrote the paper.

## CONFLICT OF INTEREST STATEMENT

The authors declare no conflict of interest.

## ETHICS STATEMENT

The study was approved by the Ethics Committee of the Affiliated Nanjing Brain Hospital, Nanjing Medical University. All patients expressed informed consent.

## Data Availability

The data that support the findings of this study are available from the corresponding author upon reasonable request.
